# Estimating GRACE terrestrial water storage anomaly using an improved point mass solution

**DOI:** 10.1038/s41597-023-02122-1

**Published:** 2023-04-22

**Authors:** Vagner Ferreira, Bin Yong, Henry Montecino, Christopher E. Ndehedehe, Kurt Seitz, Hansjörg Kutterer, Kun Yang

**Affiliations:** 1 State Key Laboratory of Hydrology-Water Resources and Hydraulic Engineering, Hohai University, Nanjing, China; 2 School of Earth Sciences and Engineering, Hohai University, Nanjing, China; 3 Department of Geodesy Science and Geomatics, University of Concepcion, Los Angeles, Chile; 4 Griffith School of Environment & Science, Griffith University, Nathan, Australia; 5 Australian Rivers Institute, Griffith University, Nathan, Australia; 6 Geodetic Institute, Karlsruhe Institute of Technology, Karlsruhe, Germany; 7 Department of Earth System Science, Ministry of Education Key Laboratory for Earth System Modeling, Institute for Global Change Studies, Tsinghua University, Beijing, China

**Keywords:** Hydrology, Hydrology

## Abstract

The availability of terrestrial water storage anomaly (TWSA) data from the Gravity Recovery and Climate Experiment (GRACE) supports many hydrological applications. Five TWSA products are operational and publicly available, including three based on mass concentration (mascon) solutions and two based on the synthesis of spherical harmonic coefficients (SHCs). The mascon solutions have advantages regarding the synthesis of SHCs since the basis functions are represented locally rather than globally, which allows geophysical data constraints. Alternative new solutions based on SHCs are, therefore, critical and warranted to enrich the portfolio of user-friendly TWSA data based on different algorithms. TWSA data based on novel processing protocols is presented with a spatial re-sampling of 0.25 arc-degrees covering 2002–2022. This approach parameterizes the improved point mass (IPM) and adopts the synthesized residual gravitational potential as observations. The assay indicates that the proposed Hohai University (HHU-) IPM TWSA data reliably agree with the mascon solutions. The presented HHU-IPM TWSA data set would be instrumental in regional hydrological applications, particularly enabling improved assessment of regional water budgets.

## Background & Summary

The measurements of the Gravity Recovery and Climate Experiment (GRACE) mission and its follow-on (GRACE-FO) provide the means for assessing terrestrial water storage anomalies (TWSA)^1^. GRACE-derived TWSA fields have improved our understanding of changes in groundwater storage^2^, hydrological fluxes estimations^3–5^, characterization of droughts and floods^6,7^, and enhancement of land surface models^8^. GRACE-derived monthly data sets are available online, mainly as Level-1b, Level-2, and Level-3 products. The Level-3 products are the TWSA monthly grids computed from the Level-1b data (GRACE raw data) using the so-called mass concentration (mascon) solution^9–11^ or based on the Level-2 data (spherical harmonic coefficients – SHCs)^12^. At present, there are three research groups in the United States, namely the Centre for Space Research (CSR), Goddard Space Flight Center (GSFC), and Jet Propulsion Laboratory (JPL), and one in Australia at the Australian National University (AUN), providing TWSA products for the public domain based on mascon solutions^9,10,13,14^. Likewise, GRACE Tellus^12^ and Gravity Information Service (GravIS)^15^ provide users with operational TWSA land products based on SHCs. Noteworthy, GravIS^15^ TWSA land products are based on the International Combination Service for Time-variable Gravity Fields (COST-G) SHCs^16^, while GRACE Tellus^12^ provides solutions based on the SHCs from CSR^17^, German Research Center for Geosciences (GFZ)^18^, and JPL^19^. Research groups from three continents (North America, Europe, and Asia) provide SHCs data sets based on different methodologies^17–26^. The recently established COST-G generates combined monthly SHCs data using the individual solutions of different analysis centers^16^. Hence, inspired by the COST-G initiative, we are motivated to pioneer a procedure and generate publicly accessible computational routines for estimating TWSA based on SHCs data sets using the novel framework proposed by Ferreira *et al*.^27,28^.

The methodology proposed to estimate TWSA here is based on the inversion of the synthesized residual gravitational potential at GRACE satellites’ altitude parameterized by the improved point mass (IPM)^27^. The advantage of such a solution is the possibility of applying spatial or spatiotemporal constraints, which otherwise would be complex based on the synthesis of SHCs^29^. Since most users would be unable to generate the residual gravitational potential based on Level-1b data, unfiltered SHCs can be used for this purpose^30–35^. Hence, the observations consist of the synthesized residual gravitational potential at GRACE’s altitude using the SHCs provided by COST-G. The choice for the COST-G solution relies on the advantage of using the statistical properties of different solutions, resulting in a reduced noise level compared to the individual solutions^16^. The generated TWSA data comprise the monthly grids at a spatial resolution of 0.25 arc-degree covering April 2002 to May 2022^36^. The new Level-3 product generated at Hohai University (HHU), namely HHU-IPM^36^, was compared with mascon solutions provided by the CSR (CSR-M^9^), GSFC (GSFC-M^13^), and JPL (JPL-M^10^). Overall, the results of the intercomparisons show that the estimated TWSA data present a root-mean-square error (RMSE) at the same level as those of mascon solutions.

Currently, only CSR-M, GSFC-M, and JPL-M are updated regularly and available for users. These solutions are based on different regularizations, parametrizations, and constraint schemes. Consequently, intercomparisons of mascon solutions in terms of long-term trends in TWSA have shown that those for JPL-M are higher than CSR-M, up to 30%^37^. Conversely, the long-term trends based on synthesized TWSA are generally 15% lower than those based on CSR mascon^37^. Consequently, applying different TWSA products based on mascon solutions and SHCs would lead to different results and conclusions. Hence, increasing the portfolio of TWSA products based on different inversion schemes would benefit the end users. This is because the intercomparisons and overall variability among the different data sets may indicate the uncertainties of the respective TWSA products. The present study’s primary goal is to present an HHU-IPM data set containing TWSA monthly grids from April 2002 to May 2022^36^. The HHU-IPM data set has a potential reuse value among hydrologists. For instance, the proposed HHU-IPM data set^36^ was used to optimize the parameters of hydrologic model^38^, which significantly supports hydrological modeling and forecasting.

## Methods

### Input data sets

All data sets used in this study as inputs (Table 1) range from April 2002 to May 2022. The GRACE/GRACE-FO COST-G^16^ (http://cost-g.org/products/) monthly solutions are given up to degree and order 90, and truncated here at degree 60 were used. COST-G is the International Gravity Field Service (IGFS) product center under the International Association of Geodesy (IAG) umbrella. The Level-2 products consisting of monthly SHCs are generated by COST-G while combining existing solutions from its analysis centers and partner analysis centers. The analysis centers consist of the Astronomical Institute of the University of Bern (AIUB), the Center National d’Etudes Spatiales (CNES), the GFZ, and the Institute of Geodesy of the Graz University of Technology (ITSG). The COST-G’s partner analysis centers consist of the CSR and the JPL. The advantage of using the COST-G product compared with the individual ensemble members is that it effectively reduces the noise level^39^ and improves signal quality^40^. The COST-G Level-2 product covers the period from April 2002 to May 2022. The geocentric gravitational constant *GM*_E_ associated with the COST-G solution refers to the value of 3.986004415 × 10^14^  m^3^s^−2^ and the Earth’s equatorial radius *a*_E_ of 6,378,136.3 m.

**Table 1:** Data sets used.

Data	Center	Degree	Reference
COST-G	International Combination Service for Time-variable Gravity Fields	90	Jäggi *et al*.^16^
Degree-ones	Jet Propulsion Laboratory	1	Sun *et al*.^41^ & Swenson *et al*.^42^
Degree-two	Goddard Space Flight Center	2	Loomis *et al*.^43^
GIA ICE-6G-D	University of Toronto	256	Peltier *et al*.^44^ & Dahle & Murboeck^45^

The different data used to generate the HHU-IPM TWSA product.

The degree-1 and degree-2 coefficients, which represent the changes in the geocenter and the oblate shape of the geopotential, were replaced with those provided by Sun *et al*.^41^ and Swenson *et al*.^42^ (https://grace.jpl.nasa.gov/data/get-data/geocenter/), and Loomis *et al*.^43^ (https://grace.jpl.nasa.gov/data/get-data/oblateness/), respectively.

The GIA model ICE-6G-D^44^ post-processed by Dahle & Murboeck^45^ (https://dataservices.gfz-potsdam.de/gravis/showshort.php?id=escidoc:4175889) was considered and used to remove the secular trends of the GIA process on GRACE measurements. Since the GIA is given as trends of SHCs per year, we used the middle point between January 2004 and December 2009 (i.e., December 31, 2006) for computing the equivalent monthly corrections.

Additionally, the aliased signal of the S2 tide by fitting a model with a constant, linear trend, annual, semi-annual, and 161-day components was removed from the monthly residual SHCs.

### Residual gravitational potential and TWSA

The relationship between TWSA (symbolically given by Δ*r*) and the residual gravitational potential (*δV*) is given by Newton’s integral, for which one of the numerical solutions is the IPM solution as:

1 $$\delta V(r,\bar{{\rm{\varphi }}},\lambda )=G{\rho }_{{\rm{w}}}\Delta \lambda \sum _{i}\Delta {\bar{{\rm{\varphi }}}}_{i}\Delta {r}_{i}{\left[{K}_{0,0}+\frac{1}{24}({K}_{2,0}\Delta {\bar{{\rm{\varphi }}}}^{2}+{K}_{0,2}\Delta {\lambda }^{2})+{\mathcal{O}}({\Delta }^{4})\right]}_{i},$$

with a further explanation available in Ferreira *et al*.^27^. In Eq. (1), *G* is Newton’s constant of gravitation, *ρ*_w_ is the density of fresh water, $\Delta \bar{\varphi }$ and Δ*λ* denote the latitudinal and longitudinal dimensions of a grid cell *i*, and *r*, $\bar{\varphi }$ and *λ* are the geocentric distance, spherical latitude, and longitude, respectively, for the observed residual gravitational potential. The coefficients *K*_*i, j*_ appearing in Eq. (1) are computed as (Heck & Seitz^46^):

2 $${K}_{i,j}=\frac{{\partial }^{i+j}}{\partial {\bar{\varphi }{\prime} }^{i}\partial {\lambda {\prime} }^{j}}{\left.\left(\frac{{R}^{2}\cos \bar{\varphi }{\prime} }{\sqrt{{r}^{2}+{R}^{2}-2rR\cos \psi }}\right)\right|}_{(\bar{\varphi }{\prime} ,\lambda {\prime} )=({\bar{\varphi }}_{0},{\lambda }_{0})},$$

at which they are evaluated at the geometric center of a grid cell defined by ${\bar{\varphi }}_{0}$ and *λ*_0_ with *ψ* being the spherical distance between the computation point ($r,\bar{\varphi },\lambda$) and the running point ($R,{\bar{\varphi }}_{0},{\lambda }_{0}$). In Eq. (1), the fourth-order and higher terms are omitted as indicated by ﻿O(Δ^4^).

Here, the Earth’s surface is represented by an ellipsoid with a semi-major axis *a* equals to 6,378,137 m, and the first eccentricity squared *e*^2^ equals to 6.69437999014 × 10^−3^. The ellipsoidal surface was discretized into cells with dimensions Δ*φ* = 1° and Δ*λ* = 1°. Noteworthy, Eqs. (1, 2) require spherical latitudes ($\bar{\varphi }$ and ${\bar{\varphi }}_{0}$), whereas the curvilinear coordinates for an ellipsoidal surface use the geodetic latitude (*φ*). Hence, the ellipsoidal coordinates (*φ*, *λ*, *h*) were transformed into spherical coordinates ($r,\bar{\varphi },\lambda$) using an intermediate transformation to Cartesian coordinates. This means that the geocentric distance *r* and the mean Earth’s radius *R* are now latitude-dependent distances. So, *R* is now the radial distance of the points at the ellipsoidal surface, which depends on the latitude and can be used to locally approximate the ellipsoid by a sphere. This local radius is the radius of the approximating sphere.

The residual gravitational potential *δV* can be computed based on Level-1b or Level-2 data sets retrieved from GRACE’s measurements. Due to computational limitations, in this work, the residual gravitational potential was synthesized from the unfiltered Level-2 products (i.e., SHCs). Given the residual SHCs $\Delta {\bar{C}}_{nm}$ and $\Delta {\bar{S}}_{nm}$ as mentioned above, the residual gravitational potential can be synthesized using the following expression (cf. Wahr *et al*.^47^):

3 $$\begin{array}{ccc}\delta V(r,\bar{\varphi },\lambda ) & = & \frac{G{M}_{{\rm{E}}}}{{a}_{{\rm{E}}}}\mathop{\sum }\limits_{n=1}^{{n}_{\max }}{\left(\frac{{a}_{{\rm{E}}}}{r}\right)}^{n+1}{(1+{k}_{n})}^{-1}\mathop{\sum }\limits_{m=0}^{n}{P}_{nm}(\sin \bar{\varphi })\\  &  & \times [\Delta {\bar{C}}_{nm}\cos (m\lambda )+\Delta {\bar{S}}_{nm}\sin (m\lambda )]\end{array},$$

In Eq. (3), *GM*_E_ and *a*_E_ refers to the geocentric gravitational constant (3.986004415 × 10^14^ m^3^s^−2^) and the Earth’s equatorial radius (6,378,136.3 m) for the COST-G geopotential model, *n* and *m* are the degrees and orders, respectively, *P*_*nm*_ are the associated Legendre functions, and the degree-dependent load Love numbers *k*_*n*_ accounting for the instantaneous reaction of the solid Earth to change surface loads. This solution was formed by a set of monthly residual SHCs complete to a maximum degree (*n*_max_) equals to 60, and no filtering was applied. The residual SHCs were computed by subtracting the mean value regarding a temporal baseline from January 2004 to December 2009 from the respective monthly values. As in Eq. (1), Eq. (3) also requires spherical coordinates, which are computed from the ellipsoidal ones using Cartesian coordinates.

### Parameter estimation using a priori constraint

Equation (1) represents the functional mode for estimating TWSA (Δ*r*) given the residual gravitational potential *δV* as the observations. Consequently, the linear system can be expressed as^48^:

4 $${\bf{d}}\,=\,{\bf{G}}{\bf{m}}+{\bf{e}},\,{\bf{e}}\sim {\mathcal{N}}(0,{{\rm{\sigma }}}_{{\bf{d}}}^{2}{\bf{I}}),$$

where **d** is the *n* × 1 vector of the residual gravitational potentials (i.e., the “observations”), **m** is the *m* × 1 vector of the unknown TWSA (Δ*r*), **G** is the *n* × *m* coefficient matrix, a matrix with the partial derivatives with respect to the model parameters Δ*r*, the unknowns, and it is given as:

5 $${\bf{G}}=\left[\begin{array}{cccc}\frac{\partial \delta {V}_{1}}{\partial \Delta {r}_{1}} & \frac{\partial \delta {V}_{1}}{\partial \Delta {r}_{2}} & \cdots  & \frac{\partial \delta {V}_{1}}{\partial \Delta {r}_{m}}\\ \frac{\partial \delta {V}_{2}}{\partial \Delta {r}_{1}} & \frac{\partial \delta {V}_{2}}{\partial \Delta {r}_{2}} & \cdots  & \frac{\partial \delta {V}_{2}}{\partial \Delta {r}_{m}}\\ \vdots  & \vdots  & \ddots  & \vdots \\ \frac{\partial \delta {V}_{n}}{\partial \Delta {r}_{1}} & \frac{\partial \delta {V}_{n}}{\partial \Delta {r}_{2}} & \cdots  & \frac{\partial \delta {V}_{n}}{\partial \Delta {r}_{m}}\end{array}\right],$$

In Eq. (4), **e** is the *n* × 1 residual vector of the observations with zero expectation and variance ${\sigma }_{{\bf{d}}}^{2}$, and **I** is the identity cofactor matrix. However, estimating TWSA at the Earth’s surface, given the residual gravitational potential at GRACE’s orbit as observations, is an ill-posed problem. This is because there is no unique solution (an infinite number of TWSA distributions can generate the same residual gravitational potential field). This inverse problem is a typical downward continuation problem in which its computation is unstable, and a small perturbation in the observations amplifies during the continuation. Furthermore, the matrix **G** is poorly conditioned, which requires special treatment in the following derivations.

Among the possibilities to solve the mentioned problem, Han *et al*.^48^ suggested a stochastic model for the vector of unknown parameters **m** using a priori information equations formulated as

6 $${{\bf{m}}}_{0}\,=\,{\bf{m}}+{{\bf{e}}}_{0},\,{{\bf{e}}}_{0}\sim {\mathcal{N}}(0,\,{{\bf{C}}}_{m}),$$

where **m**_0_ is the *m* × 1 a priori information vector of TWSA, **e**_0_ is the *m* × 1 residual vector of the a priori information with zero expectation, and **C**_*m*_, an *m* × *m* covariance matrix for **m**. Given the Eq. (4) with the constraint of Eq. (6), the damped least squares problem in the form

7 $${\left\Vert {\bf{Gm}}-{\bf{d}}\right\Vert }^{2}+{\alpha }^{2}{\left\Vert {\bf{m}}-{{\bf{m}}}_{0}\right\Vert }^{2}=\min ,$$

can be given as^49^

8 $$\widehat{{\bf{m}}}={\left({{\bf{G}}}^{{\rm{T}}}{\bf{G}}+{\alpha }^{2}{\sigma }_{{\bf{d}}}^{2}{{\bf{C}}}_{m}^{-1}\right)}^{-1}\left({{\bf{G}}}^{{\rm{T}}}{\bf{d}}+{\alpha }^{2}{\sigma }_{{\bf{d}}}^{2}{{\bf{C}}}_{m}^{-1}{{\bf{m}}}_{0}\right),$$

where *α* is a regularization parameter (or trade-off parameter). Both the regularization parameter *α* and the error variance ${\sigma }_{{\bf{d}}}^{2}$ are unknown in Eq. (8) and can be estimated as follows^49^:

9 $${\widehat{\alpha }}^{2}{\widehat{\sigma }}_{{\bf{d}}}^{2}=\frac{{{\bf{d}}}^{{\rm{T}}}{\bf{d}}-{\widehat{{\bf{m}}}}^{{\rm{T}}}(2{{\bf{G}}}^{{\rm{T}}}{\bf{d}})+{\widehat{{\bf{m}}}}^{{\rm{T}}}({{\bf{G}}}^{{\rm{T}}}{\bf{G}})\widehat{{\bf{m}}}}{n-{\left({{\bf{m}}}_{0}-\widehat{{\bf{m}}}\right)}^{{\rm{T}}}{{\bf{C}}}_{m}^{-1}\left({{\bf{m}}}_{0}-\widehat{{\bf{m}}}\right)}$$

Of course, the solutions of Eqs. (8, 9) require an iterative scheme in which initial values for *α* and ${\sigma }_{{\bf{d}}}^{2}$ should be provided at Eq. (8) to estimate $\widehat{{\bf{m}}}$.

Without better knowledge about the covariance matrix **C**_*m*_, its elements can be computed using the analytical covariance function given as

10 $${C}_{m}(\psi )={\sigma }_{{\bf{m}}}^{2}{e}^{(-\psi /D)},$$

assuming that **m** is a weakly stationary stochastic process on the sphere. In Eq. (10), *ψ* is the the spherical distances, taken pairwise, between the elements *i* and *j*, ${\sigma }_{{\bf{m}}}^{2}$ is the variance, and *D* is the correlation distance. The analytical covariance function (10) can be used to estimate the unknowns *σ*_**m**_ and *D* taking the values of the empirical covariance function

11 $${\widehat{C}}_{m}(k\Delta \psi )=\frac{\mathop{\sum }\limits_{i,j\in {\psi }_{ij}}^{M({\psi }_{ij})}{A}_{i}{A}_{j}\Delta {r}_{i}\Delta {r}_{j}}{\mathop{\sum }\limits_{i,j\in {\psi }_{ij}}^{M({\psi }_{ij})}{A}_{i}{A}_{j}},\quad k\Delta \psi \le {\psi }_{ij} < (k+1)\Delta \psi ,\quad k=0,1,\ldots ,\frac{{\psi }_{\max }}{\Delta \psi },$$

as observations. In Eq. (11), *A*_*i*_ and *A*_*j*_ are the areas, Δ*r*_*i*_ and Δ*r*_*j*_ are the TWSA values of the individual cells *i* and *j*, *M* is the number of the pairs of points at various spherical distances *ψ*_*ij*_, Δ*ψ* is the interval of spherical distance (aka the sampling interval size, e.g., 1 arc-degree), and *ψ*_max_ is the maximum spherical distance. Noteworthy, Eq. (11) groups the respective TWSA values at *i* (the reference point) and *j* (all points within distances *ψ*_*ij*_).

Again, once the empirical covariances ${\widehat{C}}_{m}$ are determined for the respective distances *k*Δ*ψ*, they can be used in Eq. (10) to estimate *σ*_**m**_ and *D*. These parameters are estimated by solving the nonlinear least squares problem, which minimizes the sum of the squares of the residues.

The a priori information **m**_0_ used here was the synthesized TWSA fields from the filtered SHCs (i.e., the filtered COST-G). Given the filtered residual SHCs $\Delta {\bar{C}}_{nm}^{{\rm{f}}}$ and $\Delta {\bar{S}}_{nm}^{{\rm{f}}}$ complete to degree 60, the synthesized TWSA is given as

12 $$\begin{array}{ccc}\Delta r\left(\bar{\varphi },\lambda \right) & = & \frac{R}{3}\frac{{\rho }_{{\rm{E}}}}{{\rho }_{{\rm{w}}}}\mathop{\sum }\limits_{n=1}^{60}\frac{2n+1}{1+{k}_{n}}\mathop{\sum }\limits_{m=0}^{n}{P}_{nm}\left(\sin \bar{\varphi }\right)\\  &  & \times \left[\Delta {\bar{C}}_{nm}^{{\rm{f}}}\cos (m\lambda )+\Delta {\bar{S}}_{nm}^{{\rm{f}}}\sin (m\lambda )\right]\end{array}.$$

In Eq. (12), *ρ*_E_ stands for the mean density of the Earth. The SHCs were de-correlated using a polynomial^50^ and smoothed using the 400-km Gaussian filter^47^ providing $\Delta {\bar{C}}_{nm}^{{\rm{f}}}$ and $\Delta {\bar{S}}_{nm}^{{\rm{f}}}$ necessary in Eq. (12). Noteworthy, the synthesized TWSA from GRACE SHCs can provide the best a priori for the parameters since it does not rely on modeling hypotheses. Furthermore, the results from Eq. (12) do not fully reconstruct the values from Eq. (3) due to the post-processing of the SHCs used on the former. Hence, it is assumed that the observations **d** in Eq. (4) and the a priori information **m**_0_ in Eq. (6) as being independent.

### Computational procedure

**Fig. 1:**
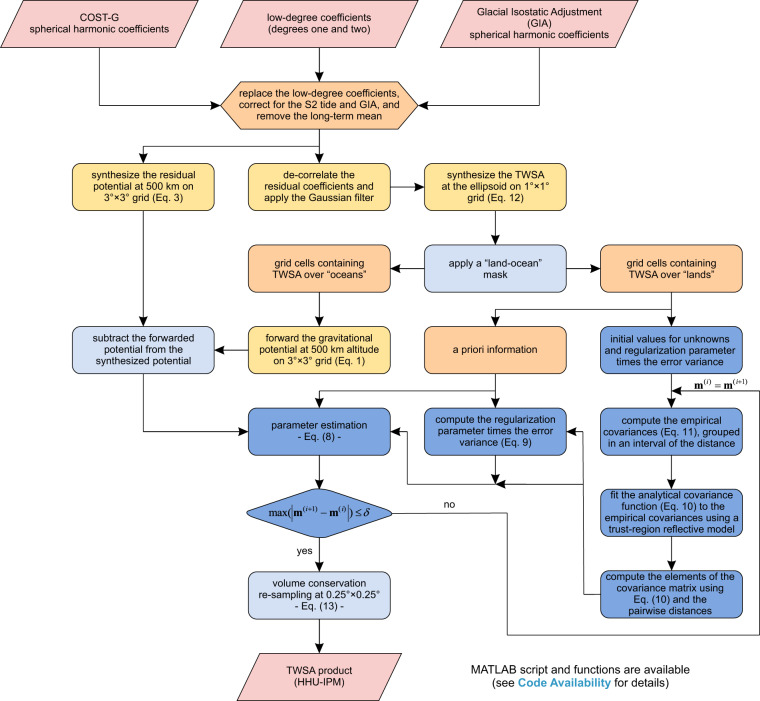
Flowchart of the HHU-IPM data generation. It shows the steps followed to produce the HHU-IPM TWSA product.

A conceptual flowchart presenting the overall computational steps is shown in Fig. 1.

The first step is to obtain the GRACE Level-2 product, here represented by the COST-G solution. The GIA effects are accounted for, the low-degree coefficients replaced, the long-term mean (2004–2009) subtracted from the respective monthly solutions, and the aliased signal of the S2 tide removed. The residual gravitational potential grids for the respective months are then synthesized at 500 km altitude at geographical bins separated by 3 arc-degree using Eq. (3). No filtering is applied to synthesize the residual gravitational potential. However, a pre-processing step for the residual SHCs at the respective epochs is necessary for synthesizing the TWSA grids as indicated in Eq. (12). The respective monthly TWSA grids with the oceans and the glaciated regions masked out are used as a priori information **m**_0_ in Eqs. (8, 9). These results in 14,438 parameters covering the lands. The gravitational potentials due to the 50,362 cells covering the oceans and glaciated regions are forwarded using Eq. (1) and then subtracted from the synthesized potentials to form the observations **d** necessary in Eqs. (8, 9). The observation vector **d** contains 5,400 elements.

In the next step, the correspondingly monthly empirical covariance functions as per Eq. (11) are computed using the a priori information **m**_0_ as an initial guess (i.e., **m**^**(**0**)**^ = **m**_0_). This is followed by estimating the respective ${\sigma }_{{\bf{m}}}^{2}$ (the variance) and *D* (the correlation distance) by solving the nonlinear least squares (nonlinear data-fitting) problem. This can be conducted using the trust-region reflective algorithm^51^. Subsequently, the respective covariance matrices **C**_**m**_, as per Eq. (10), are built using all distance pairs for the 14,438 bins. With **d**, **m**_0_, **C**_*m*_ and an initial value for the product between *α*^2^ and ${\sigma }_{{\bf{d}}}^{2}$, which should be provided, ${\widehat{{\bf{m}}}}^{(i)}$ can be estimated through Eq. (8). Here, it was adopted *α*^2^ = 1 and ${\sigma }_{{\bf{d}}}^{2}=0.002$ m^2^/s^2^ as suggested by Zhong *et al*.^49^. Next, Eq. (9) allows for estimating the product ${\alpha }^{2}{\sigma }_{{\bf{d}}}^{2}$ (${\widehat{\alpha }}^{2}{\widehat{\sigma }}_{{\bf{d}}}^{2}$) by updating ${\widehat{{\bf{m}}}}^{(i)}={\widehat{{\bf{m}}}}^{(i+1)}$ and the respective covariance **C**_*m*_ at each iteration *i*. This iterative procedure can be stopped when the maximum value of $\left|{\widehat{{\bf{m}}}}^{(i+1)}-{\widehat{{\bf{m}}}}^{(i)}\right|$ is less than a given tolerance *δ*. The tolerance value adopted in this study was 1 × 10^−6^ mm, which converged with six to ten iterations. The converged solution provides the product IPM containing the TWSA fields at 1 arc-degree resolution.

The final product IPM containing the TWSA fields is partitioned from 1 arc-degree cell to sub-cells with 0.25 arc-degrees to describe better the coastlines (or any boundaries, e.g., a river basin) and for comparison purposes against the mascon solutions. The re-sampling considers the volume conservation in the form of

13 $$\Delta r\cdot A={\sum }_{j}^{N}\left(\delta {r}_{j}\cdot {a}_{j}\right),$$

in which Δ*r* is the TWSA and *A* is the area of the original cell (e.g., 1-by-1 arc-degree), and *δr*_*i*_ and *a*_*i*_ are the respective TWSA and area values for the sub-cells *j* (the smaller cells with 0.25-by-0.25 arc-degrees) within the original cell. This can be solved through least squares at which the observation is the product Δ*r*·*A* (one observation), and the parameters are the *N* values of *δr* (16 for a cell with 1 arc-degree partitioned into sub-cells with 0.25 arc-degrees). This simple linear system with one equation and 16 unknowns can be solved by minimizing the norm of the parameters since several solutions exist to this problem. Albeit the HHU-IPM’s TWSA product is provided at 0.25-by-0.25 arc-degrees, the resolution of the data is still intrinsic to the nominal resolution of GRACE, which is 3-by-3 arc-degrees. This is compatible with the maximum degree of expansion used in Eq. (3), which the spatial (half) wavelength is given by *π*/*n*_max_. That is, Eq. (13) does not add high-frequency content (small-scale features) on the re-sampled TWSA. The re-sampling by Eq. (13) provides only spatial match-ups of the data sets for evaluation and regional averaging purposes.

MATLAB functions to perform the inversion (Fig. 1) are available for download^28^.

## Data Records

The files are freely available on figshare at 10.6084/m9.figshare.20390523. The data sets are available under the Creative Commons Licence: CC 0.

The data sets contain the GRACE monthly terrestrial water storage anomalies (TWSA) grids based on the improved point mass (IPM) solution at Hohai University (HHU), named here HHU-IPM^36^.

The HHU-IPM GRACE records for monthly TWSA values are in mm of equivalent water height for the reference period April 2002 to May 2022 in the form of a NetCDF file. They are anomalies regarding the period from January 2004 to December 2009. The data sets are provided at a spatial partitioning of 0.25-by-0.25 arc-degrees on the ellipsoid associated with the World Geodetic System 1984 (WGS84). The resolution is still GRACE’s native resolution which is 300–400 km, mainly due to the GRACE’s orbital configurations.

The variables within the file ‘HHU_IPM_GRACE_TWS_200204_202205_v04.nc’ are ‘lat’ (latitude), ‘lon’ (longitude), ‘tws’ (terrestrial water storage), and ‘time’. Latitudes and longitudes are respectively 720-by-1, and 1440-by-1 vectors in arc-degrees (decimal) north and east, respectively, and the values refer to the cells’ center. The TWSA is a 1440-by-720-by-209, at which the rows refer to the longitudes, the columns refer to latitudes, and the pages refer to the epochs (209 monthly solutions). Time is a 209-by-1 vector containing the number of days since January 1, 2002.

There are 33 missing monthly solutions, and they are 2002/06, 2002/07, 2003/06, 2011/01, 2011/06, 2012/05, 2012/10, 2013/03, 2013/08, 2013/09, 2014/02, 2014/07, 2014/12, 2015/06, 2015/10, 2015/11, 2016/04, 2016/09, 2016/10, 2017/02, 2017/07 to 2018/08, and 2018/09.

The TWSA estimations were corrected for a GIA model (Table 1) of secular trends. However, the impacts of geophysical signals due to co-seismic and/or post-seismic deformation from major offshore earthquakes were not considered. Therefore, water mass gain/loss analysis must be treated with caution in land areas near large earthquakes (e.g., 2004 Sumatra-Andaman, 2005 Nias, 2006/2007 Kuril, 2007 Bengkulu, 2009 Samoa-Tonga, 2010 Maule, 2011 Tohoku-Oki, 2012 Indian Ocean and 2013 and Okhotsk earthquakes) as listed by Han *et al*.^52^.

## Technical Validation

The HHU-IPM^36^ aims to provide users with an alternative solution for the existing GRACE TWSA data sets. However, an important question is whether the algorithm’s outcome summarized in Fig. 1 is akin to those based on mascon solutions, which are widely used at global and basin scales. The following sub-sections present ancillary data sets and the plausibility assay based on the temporal decomposition of the time series for uncertainties assessment and long-term changes of the TWSA products. Albeit mascon data were used in the comparisons, they are not avouched here as ground truth.

### Supporting data sets

#### GRACE solutions

The GRACE mascon solutions CSR-M^9^, GSFC-M^13^, and JPL-M^10^ were used for internal comparison purposes. Their main characteristics are presented in Table 2.

**Table 2:** The data sets used for comparison purposes.

Data	Center	Resolution		Reference
		Computed*	Re-sampled	
CSR-M	Centre for Space Research	~12,400 km^2^	0.25° × 0.25°	Save *et al*.^9^
GSFC-M	Goddard Space Flight Center	~12,390 km^2^	0.5° × 0.5°	Loomis *et al*.^13^
JPL-M	Jet Propulsion Laboratory	~87,003 km^2^	0.5° × 0.5°	Watkins *et al*.^10^
GravIS	German Research Centre for Geosciences	1.0° × 1.0°	—	Boergens *et al*.^15^
Basins	Global Runoff Data Centre	—	—	GRDC^57^

The GRACE solutions used for comparisons are the mascon data sets at CSR, GSFC and JPL, and GravIS at GFZ based on the COST-G Level-2b data. The GRDC’s major river basins of the world. (*) The parameters of the respective mascon products were estimated at compartments with that area values.

The mascon solutions provided by CSR, in its Release 06, version 2 (Save *et al*.^9^) were downloaded from http://www2.csr.utexas.edu/grace/, GSFC, in its Release 06, v02.4 (Loomis *et al*.^13^) were retrieved from https://earth.gsfc.nasa.gov/geo/data/grace-mascons, and JPL, in its Release 06 (cf. Watkins *et al*.^10^) were collected from https://grace.jpl.nasa.gov/. Furthermore, version 0004 (V. 0004) of the Gravity Information Service (GravIS)^15^ TWSA monthly solutions retrieved from http://gravis.gfz-potsdam.de/home were used for comparison purposes. The GravIS Level-3 data sets are based on COST-G Level-2b^53^. These products are based on the same correction model for GIA (Table 1) contributions.

The three mascon solutions, CSR-M, GSFC-M, and JPL-M, are given at a sampling of 0.25, 0.5, and 0.5 arc-degrees (Table 2). Nevertheless, their respective resolutions during the inversion are equivalent to an equal-area spherical cap corresponding to a 3 arc-degree (central angle, “diameter”) at the equator for JPL-M, and an equal-area hexagonal tile for CSR-M and an equal-area quadrangle cell for GSFC-M equals to a 1 arc-degree cell at the equator (approx. 12,400 km^2^). Noteworthy, the nominal resolution of GRACE is approximately 330 km, which is mainly constrained by the satellites’ altitude (400–500 km). However, Vishwakarma *et al*.^54^ pointed out that basins with an area of approximately 63,000 km^2^ (approx. 250 × 250 km) could be resolved with an overall error of about 20 mm. Nevertheless, a lower resolution could be possible depending on the amplitude of the TWSA signal^55^ and/or processing strategy^56^. The GSFC-M and JPL-M were re-sampled from 0.5 arc-degrees to 0.25 arc-degrees using Eq. (13), that is, conserving the volume of the original cells (0.5-by-0.5 arc-degrees). Likewise, the GravIS TWSA solutions were re-sampled from 1.0 arc-degree to 0.25 arc-degrees through Eq. (13). Noteworthy, the re-sampling does not improve (or deteriorate) the TWSA fields.

#### Selected river basins

The river basins’ delineations were downloaded from the Global Runoff Data Centre (GRDC, https://www.bafg.de), and further details can be found in reference^57^. Only 120 river basins with areas equal to or larger than 100,000 km^2^ were considered and divided into size classes. The size classes include large basins, those with areas equal to or larger than 1,000,000 km^2^ (basins 001-022), medium-size basins, those with areas equal to or larger than 360,000 km^2^ and less than 1,000,000 km^2^ (basins 023–050), and small basins those equal to or larger than 100,000 km^2^ to less than 360,000 km^2^ (basins 051–120). Noteworthy, the definition of large, medium, and small catchments did not consider the hydrological processes of the individual basins, and thereby the classification is just used in the context of the present work and GRACE’s resolution.

### Uncertainties in GRACE TWSA

The direct technical validation of TWSA products is impossible due to the nonexistence of ground-based measurements. Early studies have proposed alternative ways to evaluate different GRACE solutions^37,58–60^. In this study, the assessment suggested by Groh *et al*.^60^ was undertaken to evaluate the HHU-IPM TWSA product. Firstly, the residuals computed as the differences between the original and the detrended and de-seasoned series were smoothed using a 13-month moving average. Secondly, the difference between original and smoothed residuals provided the high-pass filtered residuals. This assessment considers only the monthly solutions from January 2004 to December 2010. The choice for this particular period for the evaluation relies on the fact that there are no missing months. Finally, the high-pass filtered residuals were then used to assess the noise level of the HHU-IPM solution based on the RMSE. RMSE values of 0 (zero) indicate a perfect fit^61^, while RMSE values less than half the standard deviation of the observations are considered low^62^. The RMSE was computed for the period from July 2004 to June 2010. The six first and the six last epochs were neglected due to the padding effects during the filtering step. The grid and basin scale evaluations are presented in the following sub-sections.

#### Grid-scale evaluation

**Fig. 2:**
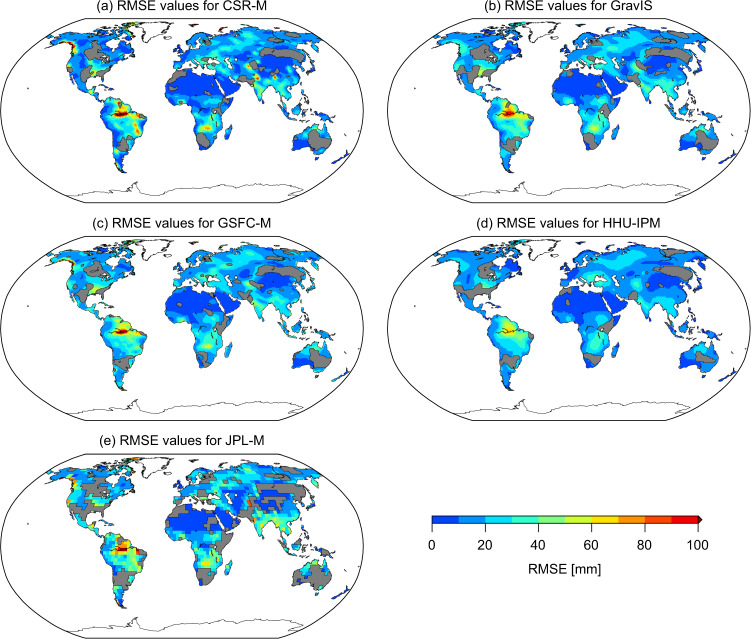
RMSE maps for the respective TWSA products. The maps show the RMSE values expressing the noise level of the respective TWSA solutions between July 2004 and June 2010 for the CSR-M (**a**), GravIS (**b**), GSFC-M (**c**), HHU-IPM (**d**), and JPL-M (**e**) solutions. The areas in grey indicate the cells at which the RMSE values of GRACE solutions are equal to or larger than half of the standard deviation of the observations.

The RMS values of the high-pass filtered residuals (i.e., RMSE) are shown in panels (a)-(e) in Fig. 2 for CSR-M, GravIS, GSFC-M, HHU-IPM, and JPL-M, respectively.

Overall, the magnitude of the RMSE values over the continents presents a spatial pattern following the TWSA variability (Fig. 2) for the five solutions. The global patterns of the RMSE values for the respective mascon solutions (CSR-M, GSFC-M, and JPL-M) and GravIS are similar to those of HHU-IPM (Fig. 2). The area-weighted average of the RMSE values provides a mean value of 16.81 mm for the HHU-IPM (Fig. 2e). The area-weighted RMSE values are 19.54 mm, 21.22 mm, and 23.16 mm for CSR-M, GSFC-M, and JPL-M, respectively. For the GravIS solution, the area-weighted RMSE value is 19.88 mm. HHU-IPM slightly overperforms CSR-M, GSFC-M, and JPL-M by approximately 14.0%, 20.8%, and 27.4%, respectively. Regarding the GravIS solution, HHU-IPM overperforms it by about 15.4%. Overall, GravIS solution outperforms GSFC-M and JPL-M, and falls behind CSR-M by approximately 1.7%. Figure 2 also depicts the grid cells at which the RMSE values are equal to or larger than half of the standard deviation of the respective TWSA time series. Notably, the JPL-M solution presents the largest number of cells at which noises dominate the signal. The area-weighted RMSE values considering only the cells with significant TWSA series in common for all solutions, the lowest value equal to 16.71 mm is seen for the HHU-IPM, which equals to an improvement of 17.4%, 17.2%, 25.2%, and 40.0% regarding the CSR-M, GravIS, GSFC-M, and JPL-M, respectively.

Nevertheless, evaluation at a regional and or local scale could provide different conclusions. For example, over areas with minimal mass variations, such as the Sahara Desert, the root-mean-square (RMS) of the detrended TSWA series is 4.79 mm for JPL-M, which outperforms CSR-M, GravIS, GSFC-M, and HHU-IPM by 117.0%, 152.8%, 173.6%, and 162.3%, respectively. (The evaluation over the Sahara Desert considered TWSA data within the region between the longitudes 3°W to 9°E and latitudes 24°N to 28°N, equivalent to 522,094.5 km^2^.) For a portion of the Gobi Desert delineated by the longitudes 100°E to 105°E and latitudes 40°N to 45°N (227,992.3 km^2^), the RMS of the TWSA series is 6.36 mm for JPL-M. This figure is equivalent to an improvement of 37.7%, 81.4%, 115.3%, and 209.0% of JPL-M regarding the CSR-M, GravIS, GSFC-M, and HHU-IPM, respectively. The performance of JPL-M using areas with minimal TWSA variations might be influenced by the geophysical model-based a priori variance approach used in the inversion^11^. Overall, all solutions performed well as analyzed by the low variability in the TWSA series with a noise level of less than 20 mm and less than 25 mm based on the temporal decomposition.

#### River-basin scale evaluation

**Fig. 3:**
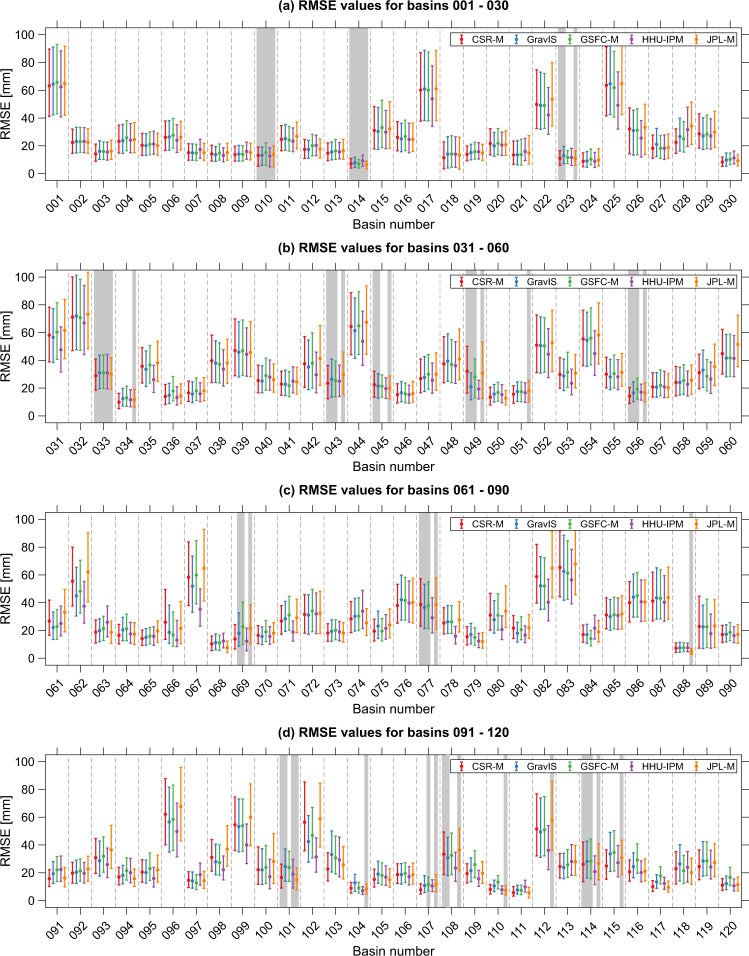
Basin-scale comparison presenting the RMSE values of the respective TWSA solutions between July 2004 and June 2010 for 120 watersheds. The filled circles of the error bars show the median of the RMSE values based on 1,000 samples, and the upper and lower caps represent the confidence intervals. The ascending basin number indicates the descending area size, e.g., basin 001 refers to the Amazon Basin and 120 to the Severn River basin. The grey rectangles indicate the basins at which the RMSE values of GRACE solutions are equal to or larger than the standard deviation of the observations.

Basin-level time series analysis was carried out over the 120 largest river basins (those with areas equal to or larger than 100,000 km^2^). Further details about the basins can be found in Table 2 and ref. ^57^. The RMSE values of the high-pass filtered residuals were computed for the respective basins’ time series. Additionally, bootstrapping^63^ was implemented to estimate confidence and significance for the RMSE values for the respective basins and solutions using 1,000 re-samplings. Figure 3 summarizes the RMSE values using the median (filled circles with different colors for the individual solutions) and the corresponding confidence intervals at 95% (the upper and lower caps).

**Fig. 4:**
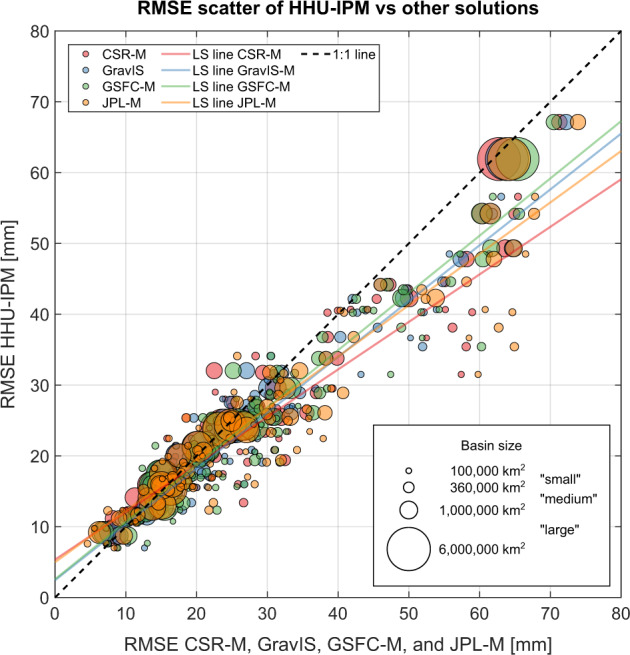
Scatter plot of the RMSE values of HHU-IPM vs those for CSR-M, GravIS, GSFC-M, and JPL-M. The RMSE values are those summarized in Fig. 3 for the respective solutions.

Uncertainties in basin-scale TWSA series are almost identical for the GRACE solutions (CSR-M, GravIS, GSFC-M, HHU-IPM, and JPL-M) as summarized in Figs. 3, 4. The HHU-IPM averaged median, considering the 120 basins’ area, gives a value of 26.00 mm (Fig. 3). This figure shows a slight outperformance of HHU-IPM in terms of the uncertainties of CSR-M, GravIS, GSFC-M, and JPL-M by approximately 3.8% (27.03 mm), 5.0% (27.37 mm), 7.4% (28.08 mm), and 8.2% (28.32 mm), respectively. Despite these slight differences (<10%), the results indicate no systematic error among the solutions. Among the mascon solutions, CSR-M generally outperforms GSFC-M and JPL-M in global and basin scales (Figs. 2, 3). However, 21 basins, indicated by the shaded rectangles in Fig. 3, present RMSE values equal to or larger than the standard deviation of the respective TWSA time series for the JPL-M solution. This characterizes relatively high RMSE values, meaning noises dominate the TWSA series over the respective basins. For the other solutions, this figure is 12, 13, 10, and 4 for CSR-M, GravIS, GSFC-M, and HHU-IPM (Fig. 3).

Similar values for the median of the RMSE values of the GRACE solutions are seen for the large basins (001–022) as depicted in Fig. 4. That is, GRACE solutions are more consistent among them for large basins. The area-weighted average for the 22 largest basins results in 25.87 mm for HHU-IPM (Fig. 3a). This shows the outperformance of approximately 1.5%, 4.2%, and 3.2% regarding the uncertainties of GravIS (26.26 mm), GSFC-M (26.99 mm), and JPL-M (26.73 mm), respectively. Conversely, HHU-IPM underperforms CSR-M by approximately 0.3% (25.79 mm). Likewise, the summary of the median values for the medium-size basins (023–050) is 27.34 mm for HHU-IPM, which outperforms CSR-M (29.78 mm), GravIS (30.06 mm), GSFC-M (30.49 mm), and JPL-M (31.56 mm) by approximately 8.2%, 9.1%, 10.3%, and 13.4%, respectively. Regarding the summary of the median for the small-size basins (051–120), HHU-IPM outperforms CSR-M (27.87 mm), GravIS (27.80 mm), GSFC-M (28.86 mm), and JPL-M (29.84 mm) by 11.8%, 11.6%, 14.9%, and 17.7% with an RMSE equals to 24.57 mm. Furthermore, the scatter of the RMSE values over small basins (100,000 – 360,000 km^2^) is relatively larger regarding the other scales (Fig. 4).

**Fig. 5:**
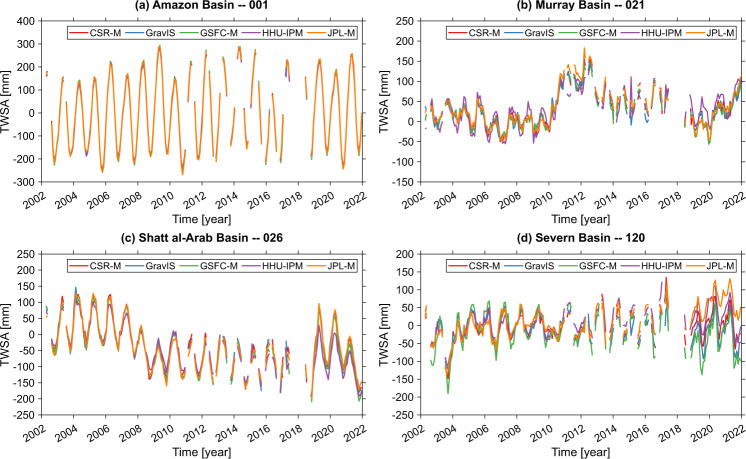
Examples of basin-scale time series. Basin averaged TWSA series derived from CSR-M, GravIS, GSFC-M, HHU-IPM, and JPL-M, solutions for (**a**) Amazon River basin – 001, (**b**) Murray River basin – 021, (**c**) Shatt al-Arab River basin – 026, and (**d**) Severn River basin – 120.

Be it grid- or basin-scale evaluation, the performance values summarized in Figs. 2, 3 only highlight that the five solutions (CSR-M, GravIS, GSFC-M, HHU-IPM, and JPL-M) agree well among them without systematic differences (Fig. 3). This also highlights that the high-pass filtered residuals of the five solutions are not affected by interannual signals and or signals with frequencies higher than semi-annual. (Or they are impacting the RMSE estimations in the same amount for the five solutions, which deserves further investigation.) Hence, the variability among the five solutions, as summarized in Figs. 2–4, may suggest that the observed uncertainties of GRACE TWSA lie within an acceptable range for hydrological applications^37^. Consequently, uncertainties in GRACE solutions based on SHCs (e.g., HHU-IPM and GravIS) appear to be similar to those based on mascon approaches in terms of noise levels (Figs. 2–4). For the sake of example, Fig. 5 shows the TWSA time series for four river basins from the largest one (Amazon Basin, Fig. 5a) to the smaller one (Severn Basin, Fig. 5d).

Overall, there is a good agreement among the five solutions and, as expected, over a large basin located at humid climate zone, GRACE products provide almost the same solution (Fig. 5a, see also Fig. 3). Conversely, differences are seen over the Severn Basin (basin n. 120), the smallest basin analyzed here (Fig. 5d). For instance, the lowest and the highest amplitudes are seen for the GSFC-M and JPL-M solutions with the other series lying between them. However, slight differences are seen over larger basins located at the semiarid climate zones as Murray Basin (Fig. 5b) and the Shatt al-Arab Basin (Fig. 5c). For these basins, the RMSE values correspondingly 13.44, 13.67, 13.69, 15.93 and 14.79 mm, and 31.96, 30.69, 31.02, 25.50 and 33.22 mm for CSR-M, GravIS, GSFC-M, HHU-IPM, and JPL-M (Fig. 3). Figure 5 also indicates that the respective GRACE solutions provide the general long-term trends over the Amazon Basin (Fig. 5a). However, the magnitude of the trends might differ among the various solutions over smaller basins across different climate zones (Fig. 5b–d).

### Long-term trends in GRACE TWSA

**Fig. 6:**
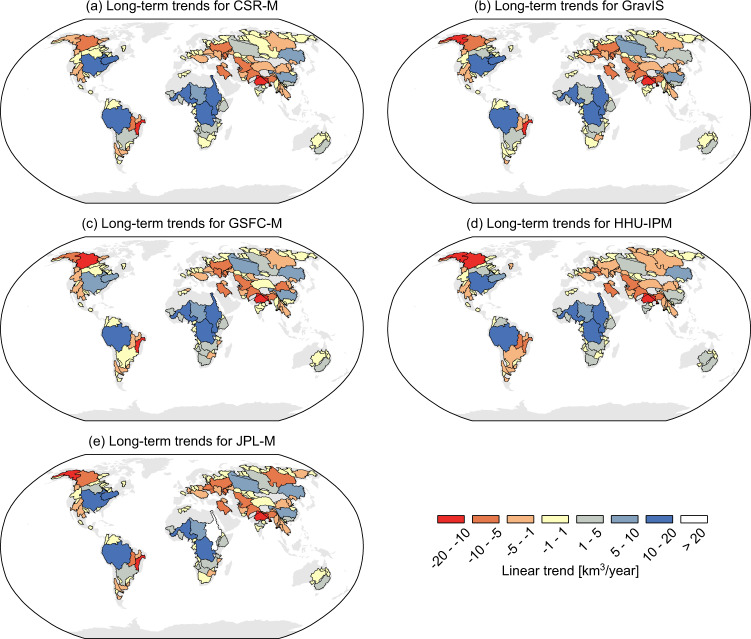
Maps showing the long-term trends of basin-scale TWSA for the respective GRACE products in km^3^ per year between Apr 2002 and Dec 2021. Panel (**a**) contains the trend values for the CSR-M solution, panel (**b**) contains the trend values for the GravIS solution, panel (**c**) contains the trend values for the GSFC-M solution, panel (**d**) depicts the long-term trends for the HHU-IPM solution, and panel (**e**) shows the long-term trends for JPL-M solution.

The long-term trends in GRACE TWSA were estimated for all solutions from April 2002 to December 2021 (common period for all solutions) over the 120 basins. Figure 6 summarizes the long-term trends for all 120 basins.

Overall, the maps with basin-level trends show similar results among the five different solutions through most of the 120 river basins. A notable difference is seen over the Yukon River basin (basin n. 028), located between the Yukon Territory in Canada and Alaska in the United States, at which HHU-IPM (Fig. 6d) shows a TWSA decrease of about −16.44 km^3^/year, whereas this figure is −3.47, −11.32, −5.59, and −15.18 km^3^/year for CSR-M, GravIS, GSFC-M, and JPL-M, respectively. Indeed, the differences between the median of the RMSE values for basin 028 are also seen in Fig. 2a, at which HHU-IPM and JPL-M present the same noise level. Figure 5d depicts a wet trend for Nile Basin (003), Africa, based on JPL-M of about 20.59 km^3^/year. Conversely, HHU-IPM presents the lowest rate amounting to 13.19 km^3^/year (Fig. 5d). The other three solutions (Fig. 5a–c) present almost the same volume change (17.23–16.77 km^3^/year). Likewise, long-term trends of TWSA based on GSFC-M show a wet trend of 9.57 km^3^/year for the Mississippi Basin (004), United States (Fig. 5c), and slightly higher values (10.45 – 13.74 km^3^/year) for the other solutions. Notably is also the linear trend depicted by HHU-IPM over the Sao Francisco River basin (038), Brazil, with a value of −6.91 km^3^/year (Fig. 5d). For this basin, the three mascon solutions and GravIS lie within the range −10.52 (CSR-M) to −10.08 km^3^/year (GSFC-M).

The GRACE solutions agree regarding the trends (wet/dry) of the long-term changes in TWSA for all river basins providing the same signal (positive/negative trend) for the individual basins. For instance, the net long-term change computed as the sum of the trends for the large-size class basins (001-022) for the respective solutions gives 64.72 km^3^/year for HHU-IPM, and 73.88, 76.48, 67.95, and 81.41 km^3^/year for CSR-M, GravIS, GSFC-M, and JPL-M, respectively. While all solutions depicted a positive net volumetric long-term change for the large-size basins, these figures are negative for the medium- and small-size basins. For instance, for all medium-size basins (023–050), the values are −65.00, −66.93, −72.40, −66.50, and −81.23 km^3^/year for CSR-M, GravIS, GSFC-M, HHU-IPM, and JPL-M, respectively. For the small-size basins (051–120), the net volumetric long-term change is −21.49, −30.14, −33.63, −19.44, and −25.88 km^3^/year for CSR-M, GravIS, GSFC-M, HHU-IPM, and JPL-M, respectively. To summarize, the sum of the volumetric changes for all basins for the respective solutions is −12.61, −20.59, −38.08, −21.22, and −25.70 km^3^/year for CSR-M, GravIS, GSFC-M, HHU-IPM, and JPL-M, respectively. So, differences can reach up to 200%, taking the CSR-M as the comparison baseline.

## Usage Notes

The TWSA data records are fundamental to studying regional/global changes in the hydrological cycles. HHU-IPM TWSA data set generated in this study^36^ can be used for various applications or studies. The HHU-IPM’s data sets can be instrumental for hydrologists in characterizing droughts^64^ and floods^65^, assimilating into hydrological models^66^, calibrating hydrological model^38^, groundwater assessment^2^, and much more^1^.

A simple MATLAB script, named “read_hhu_ipm_tws.m”, is provided alongside the data sets^36^. The script shows how to read the NetCDF file and extract the TWSA grids^36^.

## Data Availability

A MATLAB script and the necessary functions used for processing the GRACE data, as summarized in Fig. 1, are freely available for downloading on figshare at the following link: 10.6084/m9.figshare.20524035^28^. An example is provided to help the users with the overall steps. Nevertheless, due to computational limitations, the example is limited to 3-by-3 arc-degree resolution for observations and 1 arc-degree for parameters over the land areas. If desired, those with more robust computational resources can use inputs with a finer resolution. The users can use the available codes^28^ to process the data sets themselves if a more extended period is desired. This depends on the availability of the Level-2 products (e.g., COST-G solution). Alternatively, the users could use Level-2 products from different processing centers if desired.
